# Nanostructured Polymer-Dispersed Liquid Crystals Using a Ferroelectric Smectic A Liquid Crystal

**DOI:** 10.3390/molecules29204837

**Published:** 2024-10-12

**Authors:** Masaki Yamaguchi, Hiroyuki Matsukizono, Yasushi Okumura, Hirotsugu Kikuchi

**Affiliations:** 1 Interdisciplinary Graduate School of Engineering Sciences, Kyushu University, 6-1 Kasuga-Koen, Kasuga 816-8580, Fukuoka, Japan; yamaguchi.masaki.008@s.kyushu-u.ac.jp; 2 Institute for Materials Chemistry and Engineering, Kyushu University, 6-1 Kasuga-Koen, Kasuga 816-8580, Fukuoka, Japan; hmatsukizono@cm.kyushu-u.ac.jp (H.M.); okumura@cm.kyushu-u.ac.jp (Y.O.)

**Keywords:** polymer-dispersed liquid crystals, memory effect, birefringence, molecular orientation

## Abstract

Nanostructured polymer-dispersed liquid crystals (nano-PDLCs) are transparent and optically isotropic materials in which submicron-sized liquid crystal (LC) domains are dispersed within a polymer matrix. Nano-PDLCs can induce birefringence by applying an electric field (*E*-field) based on the reorientation of the LC molecules. If nano-PDLCs are utilized as light-scattering-less birefringence memory materials, it is necessary to suppress the relaxation of the LC molecule orientation after the removal of the *E*-field. We focused on the ferroelectric smectic A (SmA) phase to suppress the relaxation of LC molecules, owing to its layered structure and high viscosity. Although nano-PDLCs require a strong *E*-field to reorient their LC molecules because of the anchoring effect at the LC/polymer interface, the required field strength can be reduced using a ferroelectric smectic A (SmA_F_) LC with a large dielectric constant. In this study, we fabricated a nano-PDLC by shining an ultraviolet light on a mixture comprised an SmA_F_ LC, photocurable monomers, and a photo-initiator. The electro-birefringence effect was evaluated using polarizing optical microscopy. After the removal of the *E*-field, an enhanced memory effect was observed in the sample using SmA_F_ LC compared with nematic LC-based nano-PDLCs.

## 1. Introduction

Polymer-dispersed liquid crystals (PDLCs) are film-like solid composite materials comprising phase-separated liquid crystal (LC) domains and a polymer matrix. Polymerization-induced phase separation is the most common phase separation method. This method involves the irradiation of isotropic solutions of LCs, photocurable monomers, and a photo-initiator with ultraviolet (UV) light. The reorientation of LC molecules by applying an electric field (*E*-field) to PDLCs, which are electro-optical (EO)-responsive materials, has been used in practical applications [1,2,3,4,5,6,7,8,9,10,11]. PDLCs are generally opaque owing to their optical inhomogeneity, which causes transmitted light to scatter. Applying an *E-*field to a PDLC renders it optically uniform and transparent owing to its LC reorientation. Additionally, PDLCs exhibit light scattering–light transmission switching upon turning the *E-*field off and on, respectively. This characteristic facilitates their application as light-regulating materials. Transparent PDLCs (nano-PDLCs), even in the absence of an applied *E-*field, can be obtained by reducing the LC weight fraction in the raw material while increasing the UV irradiation intensity to form a phase-separated structure that is smaller than a visible wavelength. Nano-PDLCs, such as those with suppressed random light scattering, have been proposed as attractive EO-responsive materials for holographic films [12], microlenses [13], phase modulators [14,15], and display elements without view–angle dependence based on the electro-optical Kerr effect [16,17,18,19,20,21,22,23]. While general PDLCs switch between light scattering and transmission with the *E*-field off and on, respectively, nano-PDLCs are transparent materials regardless of whether an electric field is applied and are initially optically isotropic because of the random orientation of the LC molecules. However, when an *E*-field is applied, LCs’ reorientation and macroscopic birefringence are induced, resulting in an electro-birefringence effect. The EO responsivity of nano-PDLCs is also based on the reorientation of LC molecules inside the LC domain by an applied *E*-field. Because of the smaller size of the LC domains, nano-PDLCs require a stronger *E*-field than general PDLCs for their driving because of the large specific surface area of the LC/polymer interface and the strong influence of interface anchoring. High voltages are required to drive nano-PDLCs. Therefore, attempts have been made to lower the driving voltage by doping nano-PDLCs with conductive materials [20,21,22], such as low-*T*_g_ polymers [23]. Recently, we developed a transparent PDLC using a highly polar nematic (N) LC mixture, an LC molecule bearing a 1,3-dioxane skeleton (DIO) [24], and its analogs. These compounds exhibit large dielectric constant (*ε*′) values and induce birefringence at low driving voltages. The induced birefringence was partially maintained even after the removal of the *E*-field (memory effect) [25]. In a previous study, the birefringence viewed from the normal direction of the substrate was reversibly erased by switching the electrical circuit with a relay switch and applying an *E*-field in the out-of-plane direction of the substrate. Nano-PDLCs exhibit an electro-birefringence effect based on the reorientation of LC molecules; memorized birefringence (up to 50% of the induced birefringence) is also derived from the retention of their molecular orientation. The orientation memory effect of LC molecules after the removal of the *E*-field is expected to be more stable in layered structures with mechanical stability and in highly viscous smectic (Sm) LC phases; consequently, Sm LC-based memory-type PDLCs have also been reported [26,27]. In general, LC molecules in the smectic A (SmA) phase form a layered structure and are more viscous than those in the N phase. Therefore, the threshold voltage required for reorientation by the *E*-field of the SmA phase is higher than that of the N phase. However, the high elastic modulus also suppressed the relaxation of the molecular orientation after removal of the *E*-field. Therefore, we focus on SmA LCs with high dielectric constants parallel to the LC director. It has been demonstrated that specific DIO analog molecules exhibit a ferroelectric SmA (SmA_F_) phase with spontaneous polarization parallel to the LC director [28,29,30,31,32]. The relative dielectric constant of SmA_F_ LCs can reach several hundred [28,29,32]. Materials that can be driven at voltages lower than the driving voltages of conventional SmA LC-based materials while exhibiting a better retention of their molecular orientation than N LCs can be developed using SmA LC materials with greater dielectric anisotropy than that of conventional materials. Our earlier study showed that, in comparison to conventional SmA LCs, LC molecules with an ester skeleton had higher dielectric constant values (EST-4) [32]. In this study, we fabricated a transparent PDLC using an SmA_F_ LC (hereafter referred to as an EST). The structural and physical properties of the fabricated PDLC was assessed, and the impact of memory on the electro-birefringence effect was investigated.

## 2. Results and Discussion

**Figure 1:**
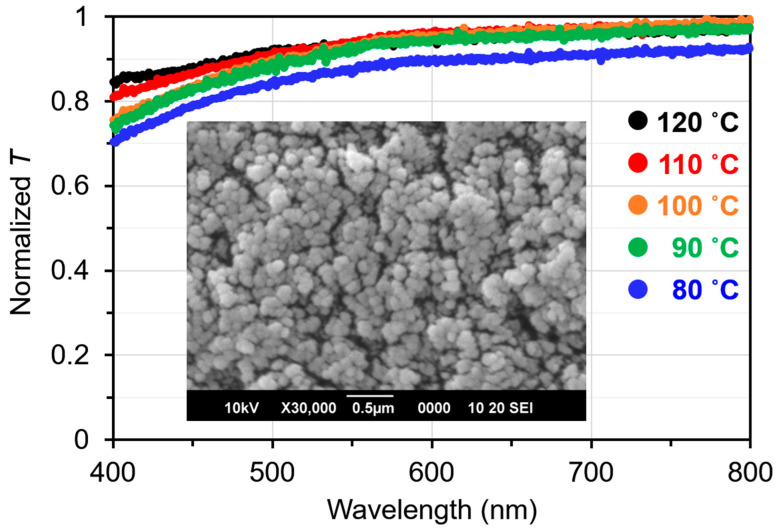
Transmittance of a PDLC normalized with a toluene-filled cell. Inset: SEM image of the PDLC of the polymer matrix.

At temperatures over 80 °C, where EST exhibited LC phases, the PDLC exhibited a higher normalized transmittance (*T* > 0.7), suggesting the formation of phase-separated structures smaller than the visible light wavelength (Figure 1). Furthermore, *T* decreases with decreasing temperature. This decrease in *T* can be attributed to the slight coarsening of the phase-separated LC domains due to the lowering of the compatibility between the EST molecules and the polymer matrix with decreasing temperature. Scanning electron microscopy (SEM) images show a phase separation of approximately 60–120 nm with a polymer ball-type morphology (Figure 1 inset and Figure S1 in the Supplementary Materials). LC droplet structures have not been observed in memory PDLCs with micrometer-sized phase-separated structures [33]. However, a similar polymer morphology was observed in the present study.

**Figure 2:**
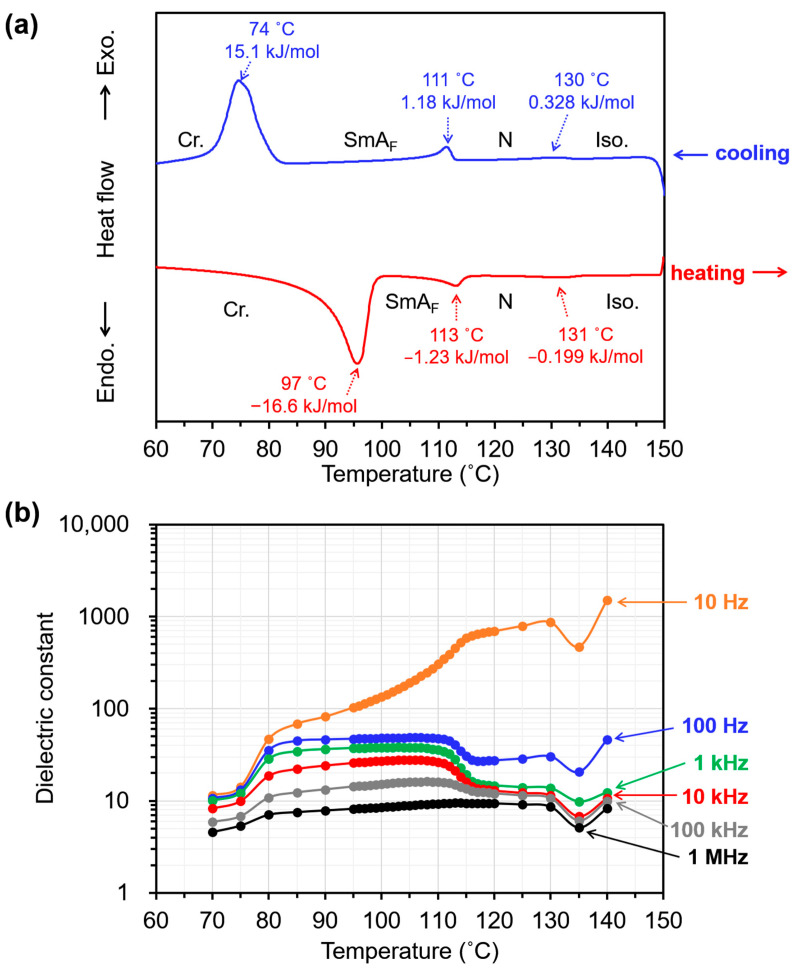
(**a**) DSC curves of the PDLC; scanning rate: 5 °C/min. (**b**) Temperature dependence of the dielectric constant (*ε*′) of the PDLC at various frequencies.

All the observed phase transition points during heating and cooling shifted toward lower temperatures than those of the bulk EST [32]. During the cooling step, exothermic peaks were observed at 130 °C (Iso. to N), 111 °C (N to SmA_F_), and 74 °C (SmA_F_ to Cr), depending on the phase transition (Figure 2a). Furthermore, the phase transition enthalpy of EST in the PDLC was lower than that of the bulk—0.328, 1.18, and 15.1 kJ/mol, which were 78, 45, and 56% of that of the bulk, respectively. The small apparent enthalpies of the phase transitions observed in the differential scanning calorimetry (DSC) measurements can be attributed to the fact that many of the ESTs dissolved in the polymer matrix or were dispersed to such a small size that they did not exhibit a phase transition. The lower transition point of Iso. to N in the PDLC compared to the bulk LC suggests a higher solubility or affinity between the LC and the polymer [34]. Furthermore, the broadening of each phase transition peak in the DSC curves suggests that the materials undergoing phase transitions are thermally destabilized and that their degree of destabilization varies widely. The phase transitions of EST occur over a broad temperature range, indicating that EST molecules adopt various states in the polymer matrix.

Next, dielectric measurements were performed to investigate the relationship between the phase transitions and the electrical properties of the PDLC. The dielectric constant (*ε*′) of the PDLC was measured within the range of 130–70 °C during the cooling process, as shown in Figure 2b. A comparison of the temperature dependence of *ε*′ at different frequencies indicates that the magnitude of *ε*′ tends to be smaller at higher frequencies, with a large change in *ε*′ between 115 and 110 °C at all frequencies. This marked temperature-dependent change in *ε*′ may be associated with the change in *E*-field responsivity due to the SmA_F_-to-N phase transition of the dispersed EST molecules in the polymer matrix. The *ε*′ value is large in the low-frequency range; however, the large apparent *ε*′ may include the conductive components of small amounts of adsorbed water in the material and small amounts of ionic conductive impurities. In addition, LC domains in an insulating polymer matrix are less likely to be subjected to an E-field, which should be considered in the low-frequency range [35]. As evident from the DSC curves in Figure 2a, the change in electrical properties at approximately 115–110 °C can be attributed to the phase transition of EST molecules.

**Figure 3:**
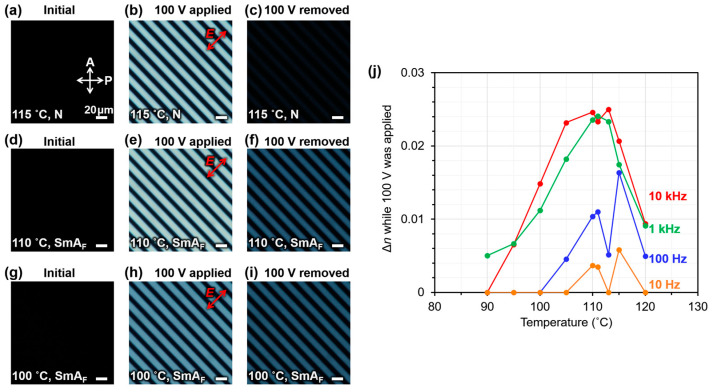
(**a**,**d**,**g**) POM images of the PDLC before applying an *E*-field at 115, 110, and 100 °C. (**b**,**e**,**h**) POM images of the PDLC during application of 100 V with a frequency of 10 kHz at 115, 110, and 100 °C. (**c**,**f**,**i**) POM images of the PDLC after removing the *E*-field at 115, 110, and 100 °C. (**j**) Temperature dependence of *E*-field-induced Δ*n* during the application of 100 V at various frequencies.

As regards Δ*n* values, Δ*n*_app_. and Δ*n*_rem_. denote the Δ*n* values during the application of an *E*-field and after the removal of an *E*-field, respectively. As shown in Figure 3a,d,g, before the application of the *E*-field, the PDLC sample does not show a Δ*n* and does not transmit light in the polarizing optical microscopy (POM) observation under crossed nicols. When a voltage of 100 V was applied at a frequency of 10 kHz, Δ*n*_app_. was induced by the *E*-field because light was transmitted between the electrodes (Figure 3b,e,h). The same procedure was also used to measure Δ*n*_app_. at frequencies of 1 kHz, 100 Hz, and 10 Hz and at various temperatures when 100 V was applied. A comparison of Δ*n*_app_. during the application of 100 V at various frequencies indicates that a larger Δ*n*_app_. was observed at higher frequencies (Figure 3j). This is contrary to the trend in *ε*′, wherein larger *ε*′ values were observed at lower frequencies. This result suggests that when 100 V is applied to the PDLC, a sufficient voltage is not applied to the EST-rich domain to reorient the EST molecules, unless it is in the high-frequency range of approximately 1 kHz or higher. If larger phase-separated structures were obtained, the PDLC would exhibit higher light scattering; however, a larger Δ*n*_app_. would be induced. The Δ*n*_app_. was almost zero during the application of 100 V in the case of a transparent PDLC with an SmA LC, 4-cyano-4′-*n*-octyloxybiphenyl (8OCB). This also confirms that PDLCs using EST are more EO-responsive than other SmA LC-based materials. After the removal of the *E*-field, Δ*n*_rem_. decreased significantly, as only a small amount of light was transmitted between the electrodes at 115 °C (Figure 3c). Meanwhile, at 110 and 100 °C, the light intensity transmitted between the electrodes was higher (brighter), indicating the retention of larger Δ*n*_rem_ values (Figure 3f,i). The retention of Δ*n*_rem_. after the removal of the *E*-field indicates that the EST molecules did not completely relax to their initial (random) molecular orientation after the removal of the *E*-field and that the molecular orientation induced by the *E*-field was partially maintained. Δ*n*_rem_. was completely erased upon heating the sample above 130 °C.

The relationship between the Δ*n* memory properties and temperatures after the removal of the *E*-field was meticulously investigated for the frequencies of 1 and 10 kHz, where better electro-birefringence effects were observed. The proportion of Δ*n* memorized after the removal of the *E-*field was defined as the memory retention rate, MRR, which is calculated as follows:

(1) $$MRR\;(\%)\;\;\equiv \;\frac{\Delta n_{rem.}}{\Delta n_{app.}}\;\times \;100.$$

**Figure 4:**
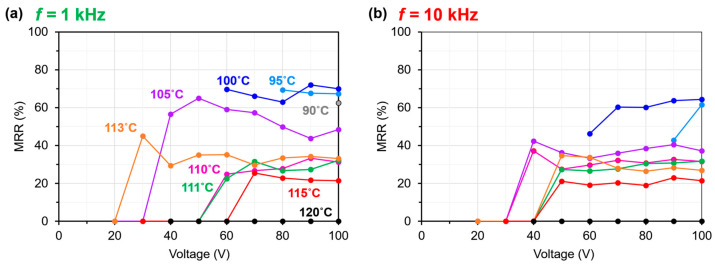
MRR in the temperature range 120–90 °C at the frequency of (**a**)1 kHz and (**b**) 10 kHz.

The temperature dependence of the MRR was less than 40% above 111 °C, indicating that EST was the N phase in the PDLC sample. However, below 100 °C, where EST is considered to have completely transitioned to SmA_F_, the MRR was more than 60%. These features were similar at the frequencies of 1 and 10 kHz (Figure 4). As evident from the DSC curve in Figure 2a, the phase transition from N to SmA_F_ proceeds within broad temperature ranges; the MRR was not likely to be high given the presence of both the SmA_F_ phase and N phase at 110 °C. At higher temperatures, the MRR was less than 40% because of the relaxation of the molecular orientation of EST to its initial orientation upon the removal of the *E*-field. In contrast, in the temperature range of the SmA_F_ phase, the elastic modulus of EST was higher than that of the N phase, and the relaxation of its molecular orientation after the removal of the *E*-field was suppressed. In addition, by using the positive-up-negative-down (PUND) method [36], currents due to polarization reversal were observed, confirming its ferroelectricity (Figure S2 in the Supplementary Materials).

Previously, we fabricated N LC-based nano-PDLCs with phase-separated structure sizes of 100–200 nm and a maximum MRR of 50% [25]. The polar monofunctional vinyl monomer NVP, which is thought to provide a polar anchoring force between the polymer interface and EST molecules, was used in this study. The anchoring effect was expected to be stronger in the nano-PDLC in this study than in the previous study because of the lower LC-constitutive fraction and smaller phase-separated structure. Furthermore, the previous work did not use NVP as the monomer. Because anchoring at the interface with the polymer is the driving source of LC reorientation after the removal of the *E*-field, a strong anchoring effect is disadvantageous for the memory effect. The high MRR (70%) in this study, despite a stronger anchoring than that in previous studies, indicates that the ability of SmA_F_ LCs to retain their molecular orientation after *E*-field removal is beneficial. Because the LC domains within the nano-PDLCs were remarkably fine, the LCs confined within them were subjected to large deformations. In the memory state of nano-PDLCs, slight deformations of the Sm layers and the presence of minor defects may be acceptable. Conversely, large layer deformations and numerous defects can lead to memory degradation [27]. The loss of approximately 30% of the Δ*n* memory is apparently due to the complex structure of the polymer and anchoring forces that distort the molecular orientation of EST near the polymer interface. This results in the inability to maintain an Sm-layered structure near the polymer, as well as partial N-like orientation ordering and enhanced relaxation. The anchoring of polymers with large polarities may be effective in maintaining their molecular orientation by retaining the ferroelectric polarization of their EST molecules. The PDLC fabricated in this study can be driven at voltages lower than those of conventional SmA LC-based materials. It can also be pinned at any Δ*n* value more efficiently than N LC-based materials, making it suitable for applications such as display elements without viewing angle dependence and electrically tunable microlenses.

## 3. Materials and Methods

### 3.1. PDLC Sample Preparation

**Figure 5:**
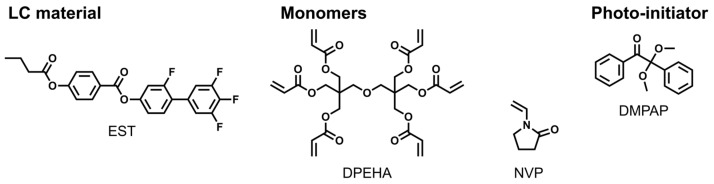
Chemical structure of substances in the PDLC precursor.

The PDLC precursor was prepared by mixing a 50 wt% (2.73 equiv.) EST as the SmA_F_ LC material (synthesized in a previous study [32]); 24.5 wt% (1.0 equiv.) Dipentaerythritol hexaacrylate (DPEHA; Tokyo Chemical Industry Co., Ltd., Tokyo, Japan) and 24.5 wt% (5.21 equiv.) of *N*-vinyl-2-pyrrolidone (NVP, Tokyo Chemical Industry Co., Ltd.) as photo-polymerizable monomers; and 1 wt% (9.21 × 10^−2^ equiv.) 2,2-dimethoxy-2-phenylacetophenone (DMPAP, Tokyo Chemical Industry Co., Ltd.) as a photo-initiator. The chemical structures of the PDLC precursors are shown in Figure 5. The hexa-functional acrylic monomer, DPEHA, was employed for curing during the initial stages of polymerization-induced phase separation. NVP, a polar vinyl monomer, was used as a solubilizer for EST with a high melting point (100 °C in bulk). The PDLC precursor was injected by capillary action into an indium tin oxide (ITO)-patterned glass cell (ISSZ-10/B707M7NSS, E.H.C. Co., Ltd., Tokyo, Japan) at 100 °C in an isotropic solution. UV light (*λ* = 365 nm peak, 50 mW/cm^2^) was then applied for 5 min at 110 °C for the photo-polymerization and phase separation to progress, yielding a transparent PDLC.

### 3.2. Light Scattering Characterization of PDLC

To investigate the structure of the resulting PDLC, a light scattering characterization of the material and the transmitted light intensity was performed using a microscope (Axio Imager.A2M, ZEISS, Oberkochen, Germany) and a compact instantaneous spectroscopic measurement unit (SA-100S-CK1, LAMBDA VISION Inc., Yokohama, Japan). The normalized transmittance (*T*) was calculated by measuring the transmitted light intensity in the wavelength range of 400–800 nm and normalizing it to the transmitted light intensity of a toluene-filled cell.

### 3.3. Scanning Electron Microscopy (SEM) Observation of Polymer Matrix

The morphology of the polymer matrix was observed using SEM. For SEM observations, the PDLC sample was soaked in dichloromethane to extract the EST molecules and dried thoroughly under vacuum. Finally, the polymer matrix was sputtered with Pt, and its morphology was characterized using SEM (Carry Scope JCM5700, JEOL Co., Ltd., Tokyo, Japan) at an accelerating voltage of 10 kV.

### 3.4. Differential Scanning Calorimetry (DSC) Measurements

Differential scanning calorimetry (DSC) of the PDLC sample was performed to investigate the phase transition behavior of EST in a fine polymer matrix. DSC curves were recorded using a differential scanning calorimeter (DSC 1 STAR^e^ System, Mettler Toledo, Greifensee, Switzerland) with a dedicated Al pan at a scanning rate of 5 °C/min.

### 3.5. Dielectric Spectroscopy of PDLC

The dielectric relaxation spectra of the PDLC were recorded in the range of 1 Hz to 1 1 MHz using an impedance/gain phase analyzer (SI 1260, Solartron Metrology, Bognor Regis, UK) at an applied voltage of 0.1 V. Cells were used with no surface orientation treatment, an ITO electrode area of 1 cm^2^, and a cell thickness of 10 µm (KSSZ-10/B107M6NSS05, E.H.C. Co., Ltd.). First, the ITO electrode’s resistance and capacitance were measured using an empty cell to correct the PDLC impedance and obtain the PDLC dielectric constants. After measuring the empty cell’s resistance and capacitance, the precursor was injected into the cell and photo-polymerized under UV irradiation.

### 3.6. Electro-Birefringence Effect

The electro-birefringence effect of the PDLC sample was measured using a polarizing optical microscope (ECLIPSE LV100 POL, Nikon, Tokyo, Japan) with a DS-Ri1 camera under crossed polarizers and by applying a sine-wave *E*-field parallel to the substrate plane with frequencies ranging from 10 Hz to 10 kHz. To determine the frequency characteristics of the electro-birefringence effect of the PDLC sample, the optical retardation (at 536 nm) of each sample was measured using a Berek compensator (Nichika Co., Ltd., Kobe, Japan). The birefringence (Δ*n*) was calculated by dividing the optical retardation by the cell thickness (10 µm).

## 4. Conclusions

A PDLC exhibiting high transparency in the visible wavelength range was fabricated using SmA_F_ LC, photopolymerizable monomers, and a photo-initiator. SEM observations revealed the formation of a fine polymer ball morphology with a size of 60–120 nm; no LC droplet morphology was observed. The phase transition behavior of the LC molecules in the PDLC was investigated using DSC, and the results suggested strong interactions between the polymer matrix and the LC molecules. Furthermore, with regard to the electro-birefringence effect, different electric birefringence responses were observed depending on the phase transition of the LCs in the PDLC. After the LCs’ transition from the N phase to the SmA_F_ phase, the proportion of memorized Δ*n* retained after the removal of the *E*-field increased, suggesting enhanced molecular orientation memory based on the mechanical stability in the SmA_F_ phase. These can be driven at voltages lower than those of conventional materials using SmA LCs. In addition, the molecular orientation memory, which was enhanced in the SmA_F_ phase, was stable after the removal of the *E*-field. These PDLCs can potentially aid the development of applications such as display elements without view–angle dependence or electrically tunable microlenses.

## Data Availability

Data are contained within the article.

## Supplementary Materials

The following supporting information can be downloaded at https://www.mdpi.com/article/10.3390/molecules29204837/s1, Figure S1: SEM images of polymer matrix at different magnifications.; Figure S2: (a) Switching current response of the PDLC while applying a triangular-wave *E*-field. (b) Hysteresis between the electric flux density and voltage measured in the SmA_F_ phase, see ref. [36].
