# Supplementary material for: Nanostructured Polymer-Dispersed Liquid Crystals Using a Ferroelectric Smectic A Liquid Crystal
Source: Molecules. 2024 Oct 12;29(20):4837. doi: 10.3390/molecules29204837 (PMC11510082; doi:10.3390/molecules29204837)
Supplement: Supplementary file 1 [file molecules-29-04837-s001.zip › molecules-3238042-supplementary.pdf]

Supporting Information

1. Scanning Electron microscope (SEM) images of the polymer matrix

SEM images of the polymer matrix of the PDLC sample after extracting EST molecules are shown in Figure S1. The area in contact with the glass substrate appears dark in the SEM image and is therefore flat, while the polymer inside appears bright, indicating a polymer ball-like uneven structure.

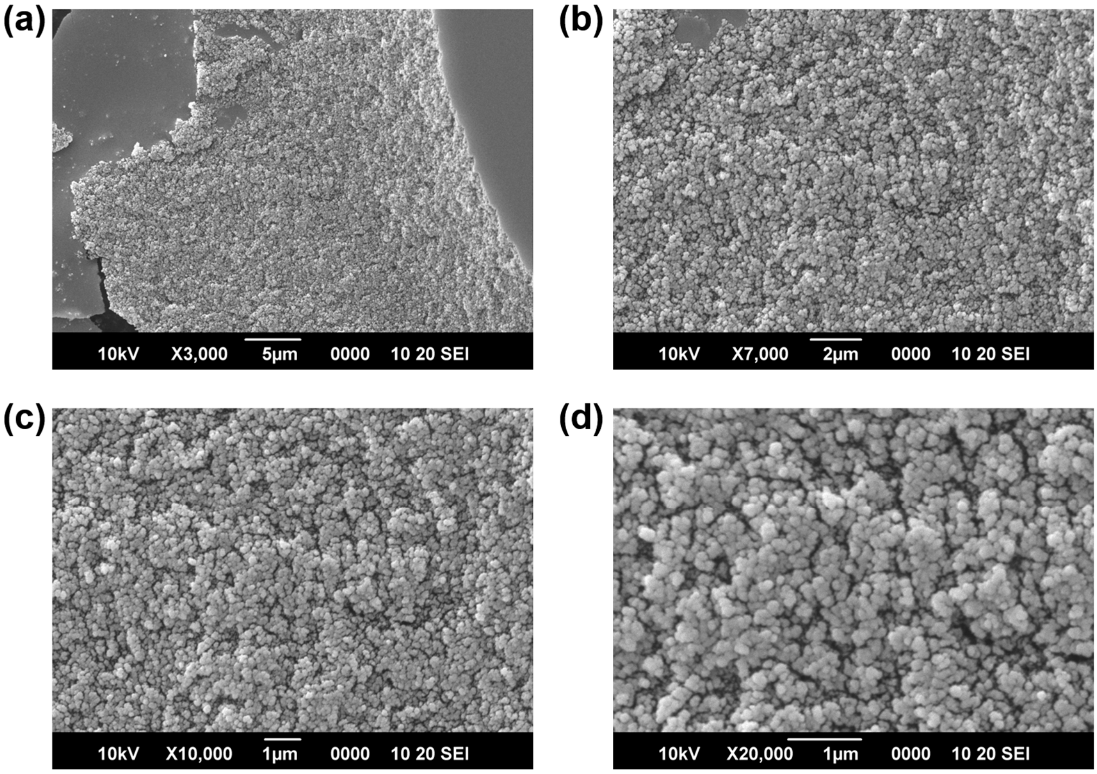

Figure S1. SEM images of the polymer matrix at different magnifications. a), b), c) and d) are multiplied by 3000, 7000, 10000 and 20000 times, respectively.

2. Switching current response of the PDLC

Switching current response of the PDLC was measured using the positive-up–negative-down (PUND) polarization measurement technique. A triangular-wave electric field (2.0 V/μm, *f* = 100 Hz) was applied to the PDLC sample and the corresponding currents were shown in Figure 2a. We used an ITO-coated cell (square ITO area, 10 × 10 mm^2, thickness: 10 μm) with no surface orientation treatment.

When a triangular-wave *E*-field was applied to the PDLC sample, currents due to polarization reversal were observed below 110 ˚C, suggesting the appearance of a ferroelectric LC phase in the PDLC. The observed current values of a few hundred nA are small, probably due to the small fraction of EST molecules that can respond to the *E*-field. Furthermore, hysteresis between the electric flux density and applied voltage was observed at temperatures where the EST is in SmA_F phase (Figure 2b). The observed current values decreased with decreasing temperature and the threshold voltage required for switching increased. An increase in the viscosity of the ESTs is possible causes. On the other hand, no currents originating from polarization reversal were observed at 115 °C, when the EST is in the paraelectric N phase.

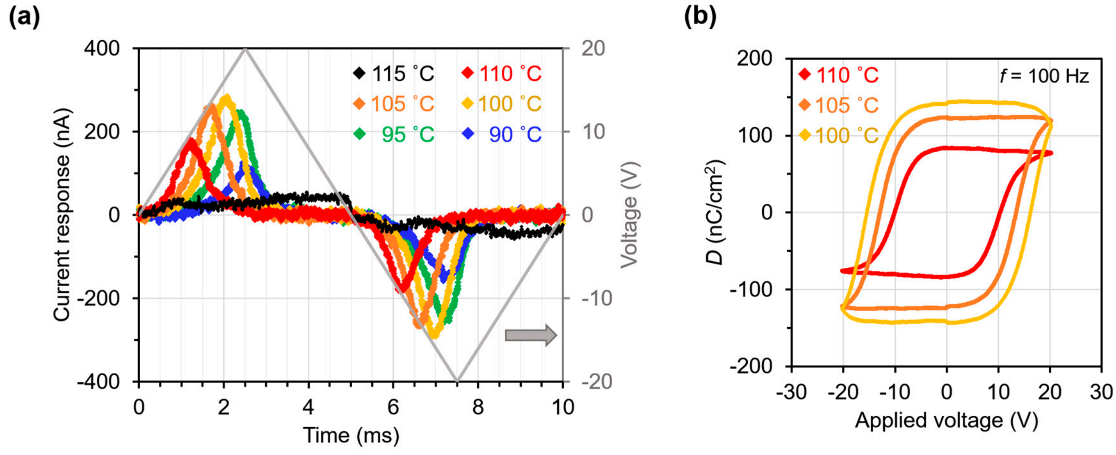

Figure S2. a) Switching current response of the PDLC while applying a triangular-wave *E*-field. b) Hysteresis between the electric flux density and voltage measured in SmA_F phase.
